# Wolverine Veterinary Medical Association

**Published:** 1901-08

**Authors:** W. W. Thorburn

**Affiliations:** Secretary


### SOCIETY PROCEEDINGS.

### WOLVERINE VETERINARY MEDICAL ASSOCIATION.

THE Wolverine Veterinary Medical Association met at Grand Rapids, Mich., July 3, 1901. The Association was called to order by the President, Dr. B. O. Johnson, of Benton Harbor, at 10.30 A.M. As the Secretary, Dr. W. W. Thorburn, had not yet arrived, Dr. William C. Wootton, by request of the President, acted as Secretary *pro tem.*

President Johnson then suggested that inasmuch as the Secretary, minutes, and programme were absent that each member present be called upon to relate his latest and most interesting case. Dr. Eugene Brown, of Lawrence, Mich., related a peculiar case of hernia occurring in a mare several days before parturition. Dr. M. Nelson, of Manistee, was the next speaker. Dr. Nelson is a Swede by birth and holds a diploma from a Swedish veterinary college. While his accent was somewhat broken, yet he soon convinced his hearers that he is an up-to-date veterinarian, relating several cases of fœtal dystokia that were of benefit as well as being very interesting to the Association. Dr. Johnson, President of the Association, related two very interesting cases of dystokia in the cow. Dr. W. C. Wootton then related a case of injury from a scythe, where the metacarpal arteries, flexor pedis perforatus and perforans were completely divided, and the suspensory ligament was partially severed.

Dr. L. L. Conkey was the next speaker, and gave a very interesting sketch of veterinary legislation in Michigan, exhibiting letters that he had received from members of the House of Representatives showing that the failure to secure favorable legislation at the last session of that body was due to the intervention of the old Michigan Veterinary Association, or, rather, to the " foreign element " that dominates the actions of that Association.

*Afternoon Session.*—The meeting was called to order by the President at 2 P.M., with Secretary W. W. Thorburn in the Chair. Dr. P. Hesseltine read a paper on " State Meat-inspection," which was discussed at some length, and Prof. W. C. Wootton, of the Grand Rapids Veterinary College, read a paper entitled " Two Cases of Equine Tuberculosis." There not being much time left for discussion, those papers were, by motion, ordered printed in our next annual report.

The Association then went into executive session, during which time considerable routine business was transacted.

The following were appointed by the President to read papers at the annual meeting, which is to be held the last Tuesday in December at Lansing, Mich. : Dr. R. S. Coleman, of Sparta ; Dr. M. Nelson, of Manistee ; Dr. B. F. Baldwin, of Rockford ; Dr. Eugene Brown, of Lawrence, and Dr. J. W. Ackerson, of Manchester, Mich.

The meeting then adjourned to meet at Lansing, Tuesday, December 30th, at 9 A.M., at a place to be designated by the Secretary.

W. W. THORBURN,
Secretary.

